# Supplementary material for: Enhanced ER proteostasis and temperature differentially impact the mutational tolerance of influenza hemagglutinin
Source: eLife. 2018 Sep 6;7:e38795. doi: 10.7554/eLife.38795 (PMC6172027; doi:10.7554/eLife.38795)
Supplement: Supplementary file 1. [file elife-38795-supp1.docx]

Supplementary File 1. Primer sequences for qPCR and HA sequencing.

| **Primer Name** | **Primer Sequence** | **Method** |
| --- | --- | --- |
| *HSP90*_fwd | GATAAACCCTGACCATTCC | qPCR |
| *HSP90*_rev | AAGACAGGAGCGCAGTTTCATAAA | qPCR |
| *HSP70*_fwd | GGAGGCGGAGAAGTACA | qPCR |
| *HSP70*_rev | GCTGATGATGGGGTTACA | qPCR |
| *HSP40*_fwd | TGTGTGGCTGCACAGTGAAC | qPCR |
| *HSP40*_rev | ACGTTTCTCGGGTGTTTTGG | qPCR |
| *RPLP2*_fwd | CGTCGCCTCCTACCTGCT | qPCR |
| *RPLP2*_rev | CCATTCAGCTCACTGATAACCTTG | qPCR |
| *ERDJ4*_fwd | CTGTATGCTGATTGGTAGAGTCAA | qPCR |
| *ERDJ4*_rev | AGTAGACAAAGGCATCATTTCCAA | qPCR |
| *BiP*_fwd | GCCTGTATTTCTAGACCTGCC | qPCR |
| *BiP*_rev | TTCATCTTGCCAGCCAGTTG | qPCR |
| *SEC24D*_fwd | AGCAGACTGTCCTGGGAAGC | qPCR |
| *SEC24D*_rev | TTTGTTTGGGGCTGGAAAAG | qPCR |
| *GRP94*_fwd | GGCCAGTTTGGTGTCGGT | qPCR |
| *GRP94*_rev | CGTTCCCCGTCCTAGAGTGTT | qPCR |
| *MATRIX*_fwd | AGATGAGTCTTCTAACCGAGGTCG | qPCR |
| *MATRIX*_rev | TGCAAAAACATCTTCAAGTCTCTG | qPCR |
| WSNHA-For | AGCAAAAGCAGGGGAAAATAAAAACAAC | PCR of HA amplicon |
| WSNHA-Rev | AGTAGAAACAAGGGTGTTTTTCCTTATATTTCTG | PCR of HA amplicon |
| Subamplicon_1_fwd | CTTTCCCTACACGACGCTCTTCCGATCTNNNNNNNNAAGCAGGGGAAAATAAAAACAACCAAA | PCR1 of library prep |
| Subamplicon_1_rev | GGAGTTCAGACGTGTGCTCTTCCGATCTNNNNNNNNCATTCTCAGAGTTTGGTGTTTCTACAAT | PCR1 of library prep |
| Subamplicon_2_fwd | CTTTCCCTACACGACGCTCTTCCGATCTNNNNNNNNTCCAGCGAGATCATGGTCCTAC | PCR1 of library prep |
| Subamplicon_2_rev | GGAGTTCAGACGTGTGCTCTTCCGATCTNNNNNNNNGGGTGATGAACACCCCATAGTAC | PCR1 of library prep |
| Subamplicon_3_fwd | CTTTCCCTACACGACGCTCTTCCGATCTNNNNNNNNTGTGAACAATAAAGGGAAAGAAGTCCTT | PCR1 of library prep |
| Subamplicon_3_rev | GGAGTTCAGACGTGTGCTCTTCCGATCTNNNNNNNNGTGTTACACTCATGCATTGACGC | PCR1 of library prep |
| Subamplicon_4_fwd | CTTTCCCTACACGACGCTCTTCCGATCTNNNNNNNNGTCCGGCATCATCACCTCAAAC | PCR1 of library prep |
| Subamplicon_4_rev | GGAGTTCAGACGTGTGCTCTTCCGATCTNNNNNNNNGTTAATGGCATTTTGTGTGCTTTTTTG | PCR1 of library prep |
| Subamplicon_5_fwd | CTTTCCCTACACGACGCTCTTCCGATCTNNNNNNNNGATCAGGCTATGCAGCGGAT | PCR1 of library prep |
| Subamplicon_5_rev | GGAGTTCAGACGTGTGCTCTTCCGATCTNNNNNNNNGAACTCAAAACACCCATTTCCGAT | PCR1 of library prep |
| Subamplicon_6_fwd | CTTTCCCTACACGACGCTCTTCCGATCTNNNNNNNNAAAAAGCCAATTAAAGAATAATGCCAAAGAA | PCR1 of library prep |
| Subamplicon_6_rev | GGAGTTCAGACGTGTGCTCTTCCGATCTNNNNNNNNGGGTGTTTTTCCTTATATTTCTGAAATCCTAATC | PCR1 of library prep |
| Universal_fwd | AATGATACGGCGACCACCGAGATCTACACTCTTTCCCTACACGACGCTCTTCCGATCT | PCR2 of library prep |
| Rnd2_rev_1 | CAAGCAGAAGACGGCATACGAGATACATCGGTGACTGGAGTTCAGACGTGTGCTCTTCCGATCT | PCR2 of library prep |
| Rnd2_rev_2 | CAAGCAGAAGACGGCATACGAGATCACTGTGTGACTGGAGTTCAGACGTGTGCTCTTCCGATCT | PCR2 of library prep |
| Rnd2_rev_3 | CAAGCAGAAGACGGCATACGAGATGCCTAAGTGACTGGAGTTCAGACGTGTGCTCTTCCGATCT | PCR2 of library prep |
| Rnd2_rev_4 | CAAGCAGAAGACGGCATACGAGATTCAAGTGTGACTGGAGTTCAGACGTGTGCTCTTCCGATCT | PCR2 of library prep |
| Rnd2_rev_5 | CAAGCAGAAGACGGCATACGAGATCTGATCGTGACTGGAGTTCAGACGTGTGCTCTTCCGATCT | PCR2 of library prep |
| Rnd2_rev_6 | CAAGCAGAAGACGGCATACGAGATAAGCTAGTGACTGGAGTTCAGACGTGTGCTCTTCCGATCT | PCR2 of library prep |
| Rnd2_rev_7 | CAAGCAGAAGACGGCATACGAGATCGTACGGTGACTGGAGTTCAGACGTGTGCTCTTCCGATCT | PCR2 of library prep |
| Rnd2_rev_8 | CAAGCAGAAGACGGCATACGAGATATCAGTGTGACTGGAGTTCAGACGTGTGCTCTTCCGATCT | PCR2 of library prep |
| Rnd2_rev_9 | CAAGCAGAAGACGGCATACGAGATCACTGTGTGACTGGAGTTCAGACGTGTGCTCTTCCGATCT | PCR2 of library prep |
| Rnd2_rev_10 | CAAGCAGAAGACGGCATACGAGATATTGGCGTGACTGGAGTTCAGACGTGTGCTCTTCCGATCT | PCR2 of library prep |
| Rnd2_rev_11 | CAAGCAGAAGACGGCATACGAGATTACAAGGTGACTGGAGTTCAGACGTGTGCTCTTCCGATCT | PCR2 of library prep |
| Rnd2_rev_12 | CAAGCAGAAGACGGCATACGAGATTGTTGACTGTGACTGGAGTTCAGACGTGTGCTCTTCCGATCT | PCR2 of library prep |
| Rnd2_rev_13 | CAAGCAGAAGACGGCATACGAGATACGGAACTGTGACTGGAGTTCAGACGTGTGCTCTTCCGATCT | PCR2 of library prep |
| Rnd2_rev_14 | CAAGCAGAAGACGGCATACGAGATTCTGACATGTGACTGGAGTTCAGACGTGTGCTCTTCCGATCT | PCR2 of library prep |
| Rnd2_rev_15 | CAAGCAGAAGACGGCATACGAGATGTGCGGACGTGACTGGAGTTCAGACGTGTGCTCTTCCGATCT | PCR2 of library prep |
| Rnd2_rev_16 | CAAGCAGAAGACGGCATACGAGATCGTTTCACGTGACTGGAGTTCAGACGTGTGCTCTTCCGATCT | PCR2 of library prep |
| Rnd2_rev_17 | CAAGCAGAAGACGGCATACGAGATAAGGCCACGTGACTGGAGTTCAGACGTGTGCTCTTCCGATCT | PCR2 of library prep |
| Rnd2_rev_18 | CAAGCAGAAGACGGCATACGAGATTCCGAAACGTGACTGGAGTTCAGACGTGTGCTCTTCCGATCT | PCR2 of library prep |
| Rnd2_rev_19 | CAAGCAGAAGACGGCATACGAGATATCCACTCGTGACTGGAGTTCAGACGTGTGCTCTTCCGATCT | PCR2 of library prep |
| Rnd2_rev_20 | CAAGCAGAAGACGGCATACGAGATAAAGGAATGTGACTGGAGTTCAGACGTGTGCTCTTCCGATCT | PCR2 of library prep |
| Rnd2_rev_21 | CAAGCAGAAGACGGCATACGAGATTAGTTGGTGACTGGAGTTCAGACGTGTGCTCTTCCGATCT | PCR2 of library prep |
| Rnd2_rev_22 | CAAGCAGAAGACGGCATACGAGATCCGGTGGTGACTGGAGTTCAGACGTGTGCTCTTCCGATCT | PCR2 of library prep |
