# Supplementary material for: Enhanced ER proteostasis and temperature differentially impact the mutational tolerance of influenza hemagglutinin
Source: eLife. 2018 Sep 6;7:e38795. doi: 10.7554/eLife.38795 (PMC6172027; doi:10.7554/eLife.38795)
Supplement: Source data 1. [file elife-38795-data1.zip › Dataset_1/HA_DMS_2018_processing/logo_plots/DMSOtemp_all_pickouts_sorted_for_logo.pdf]

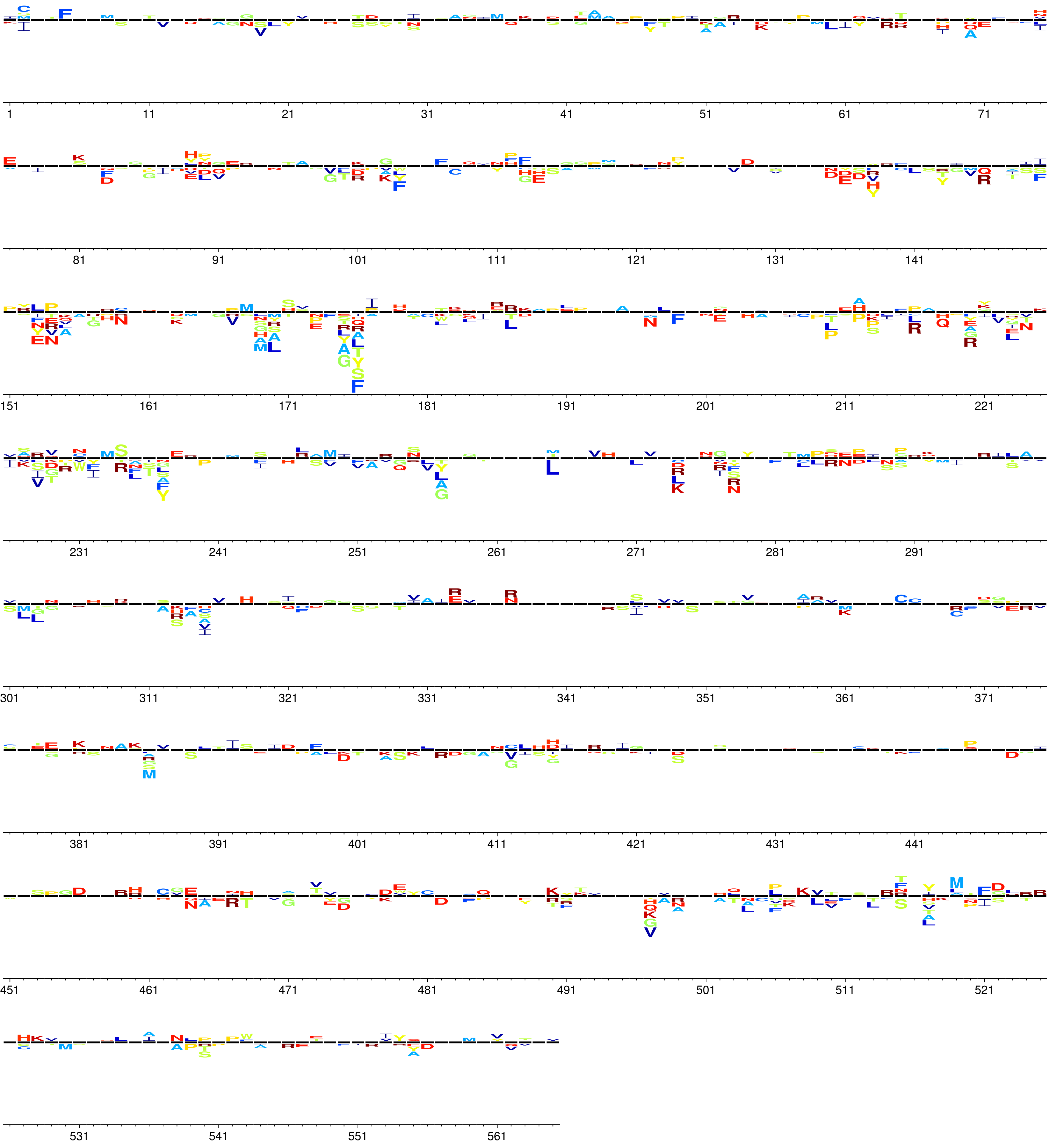
1 11 21 31 41 51 61 71
81 91 101 111 121 131 141
151 161 171 181 191 201 211 221
231 241 251 261 271 281 291
301 311 321 331 341 351 361 371
381 391 401 411 421 431 441
451 461 471 481 491 501 511 521
531 541 551 561
