# Supplementary material for: Enhanced ER proteostasis and temperature differentially impact the mutational tolerance of influenza hemagglutinin
Source: eLife. 2018 Sep 6;7:e38795. doi: 10.7554/eLife.38795 (PMC6172027; doi:10.7554/eLife.38795)
Supplement: Source data 1. [file elife-38795-data1.zip › Dataset_1/HA_DMS_2018_processing/logo_plots/XBP139_all_pickouts_sorted_for_logo.pdf]

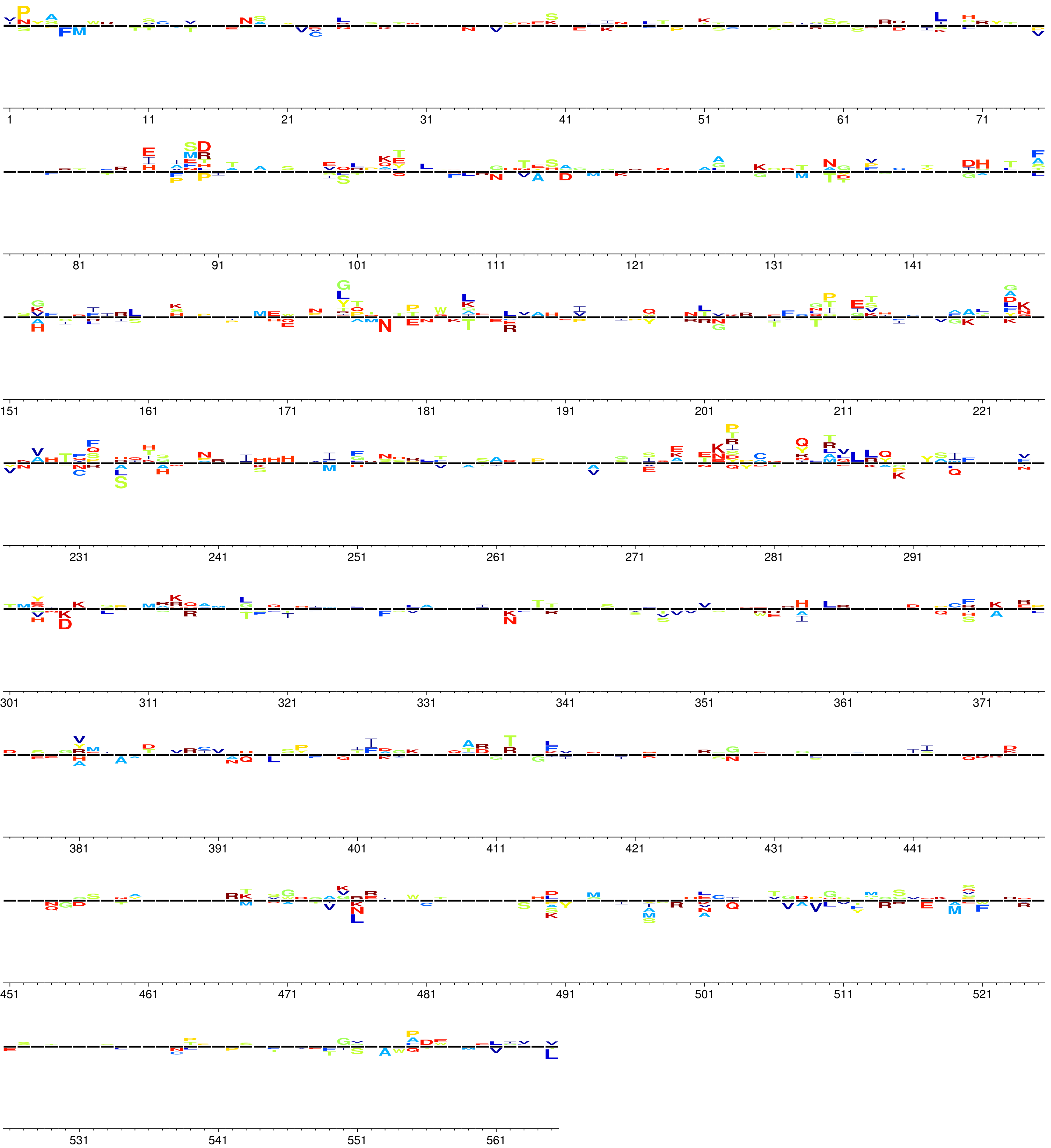
1
11
21
31
41
51
61
71
81
91
101
111
121
131
141
151
161
171
181
191
201
211
221
231
241
251
261
271
281
291
301
311
321
331
341
351
361
371
381
391
401
411
421
431
441
451
461
471
481
491
501
511
521
531
541
551
561
