# Supplementary material for: Enhanced ER proteostasis and temperature differentially impact the mutational tolerance of influenza hemagglutinin
Source: eLife. 2018 Sep 6;7:e38795. doi: 10.7554/eLife.38795 (PMC6172027; doi:10.7554/eLife.38795)
Supplement: Source data 1. [file elife-38795-data1.zip › Dataset_1/replicate-1/alignmentsummary_mutfreqs.pdf]

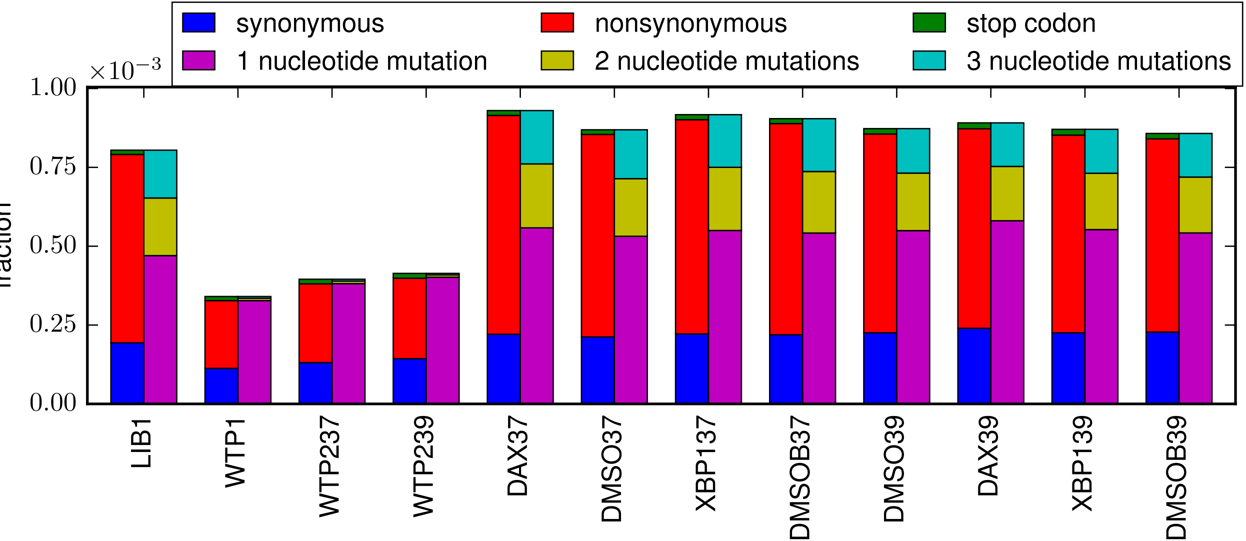

synonymous
nonsynonymous
stop codon
1 nucleotide mutation
2 nucleotide mutations
3 nucleotide mutations
$\times 10^{-3}$
1.00
0.75
0.50
0.25
0.00
fraction
LIB1
WTP1
WTP237
WTP239
DAX37
DMSO37
XBP137
DMSOB37
DMSO39
DAX39
XBP139
DMSOB39
