# Supplementary material for: Enhanced ER proteostasis and temperature differentially impact the mutational tolerance of influenza hemagglutinin
Source: eLife. 2018 Sep 6;7:e38795. doi: 10.7554/eLife.38795 (PMC6172027; doi:10.7554/eLife.38795)
Supplement: Source data 1. [file elife-38795-data1.zip › Dataset_1/replicate-2/alignmentsummary_barcodes.pdf]

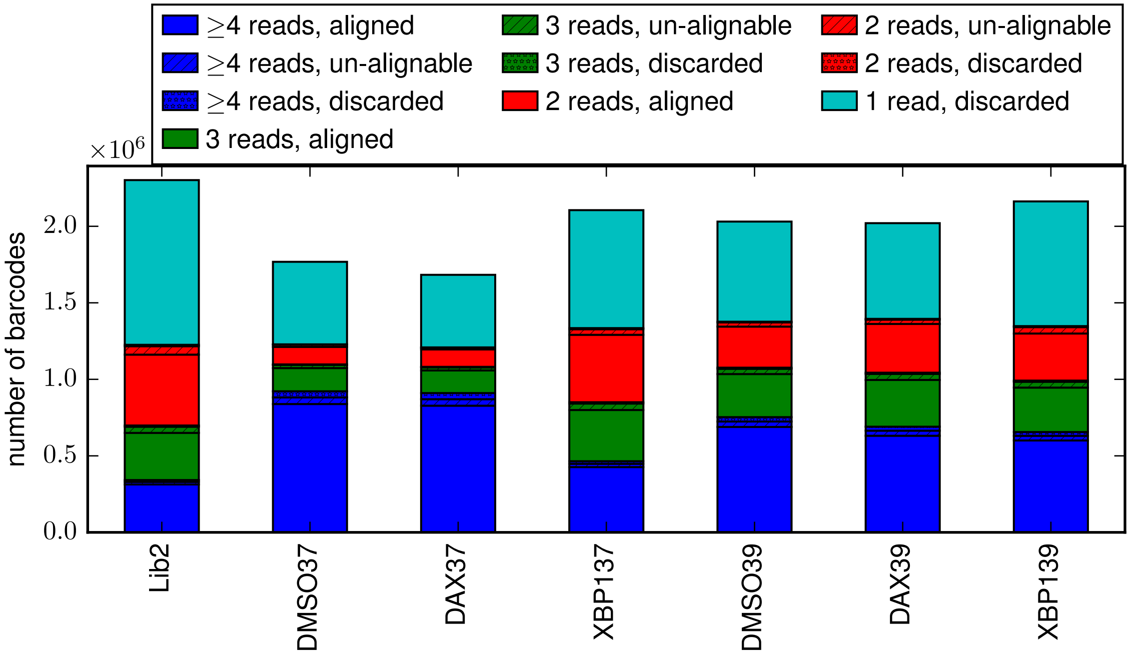
≥4 reads, aligned
≥4 reads, un-alignable
≥4 reads, discarded
3 reads, aligned
3 reads, un-alignable
3 reads, discarded
2 reads, aligned
2 reads, un-alignable
2 reads, discarded
1 read, discarded
×10^6
number of barcodes
0.0
0.5
1.0
1.5
2.0
Lib2
DMSO37
DAX37
XBP137
DMSO39
DAX39
XBP139
