# Supplementary material for: Enhanced ER proteostasis and temperature differentially impact the mutational tolerance of influenza hemagglutinin
Source: eLife. 2018 Sep 6;7:e38795. doi: 10.7554/eLife.38795 (PMC6172027; doi:10.7554/eLife.38795)
Supplement: Source data 1. [file elife-38795-data1.zip › Dataset_1/replicate-3/alignmentsummary_depth.pdf]

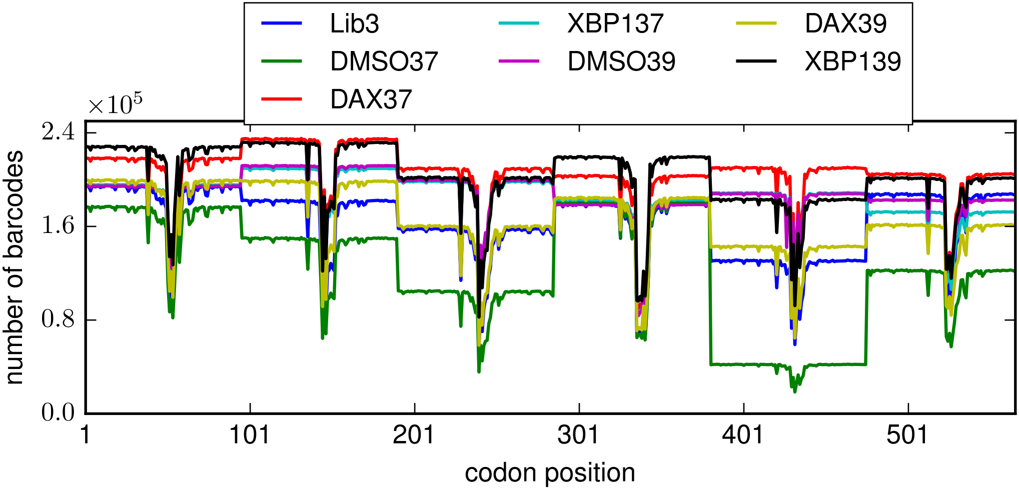

Lib3
DMSO37
DAX37
XBP137
DMSO39
DAX39
XBP139
×10^5
2.4
1.6
0.8
0.0
number of barcodes
1
101
201
301
401
501
codon position
